# Concurrent PIK3CA and IDH1 variants in facial infiltrating lipomatosis with intracranial lesions

**DOI:** 10.1016/j.gendis.2024.101324

**Published:** 2024-05-22

**Authors:** Hongrui Chen, Bin Sun, Lizhen Wang, Lei Chang, Zhang Yu, Wei Gao, Yajing Qiu, Hui Chen, Chen Hua, Xiaoxi Lin

**Affiliations:** a Department of Plastic & Reconstructive Surgery, Shanghai Ninth People's Hospital, Shanghai Jiao Tong University School of Medicine, Shanghai 200011, China; b Department of Oral Pathology, Shanghai Ninth People's Hospital, Shanghai Jiao Tong University School of Medicine, Shanghai 200011, China

Facial infiltrating lipomatosis (FIL) is a congenital disorder caused by the hyperproliferation of adipose and skeletal tissue within the facial region. Infiltration of mature adipose tissue into adjacent structures is a hallmark pathologic finding. In addition to the aesthetic implications, patients may suffer from impaired facial function, including difficulty in swallowing and breathing, sleep disturbances, and visual field displacement. FIL is associated with phosphatidylinositol 3-kinase catalytic subunit alpha (PIK3CA) variants. PIK3CA variants were detected in over 85% of FIL cases, spanning numerous tissue types. Prior research has indicated that PIK3CA hotspot variants may result in a more severe phenotype. However, identical PIK3CA variants can lead to varying degrees of phenotypic severity. This highlights the need for further exploration into the potential contribution of other pathogenic factors. In this study, we reported the co-occurrence of PIK3CA and isocitrate dehydrogenase 1 (IDH1) variants in two patients with FIL and intracranial lesions for the first time. Our study expands the genetic landscape of FIL and provides a view that variants in genes other than PIK3CA may be involved in the pathogenesis of overgrowth disorders.

Patient No.1, a 13-year-old female, presented with conspicuous facial enlargement since birth. She was the first child of non-consanguineous parents. At the age of 3, she underwent debulking surgery, followed by liposuction and lip adjustment at age 4. Unfortunately, the patient experienced recurrence. At age 6, another surgical intervention involving debulking and partial osteotomy of the zygoma and mandible was performed. The patient did not undergo any further medical interventions. Physical examination revealed facial asymmetry, left eye strabismus, and hemimacroglossia. Visible surgical scars on the lips and left cheek were observed (Fig. 1A). Computer tomography (CT) skeletal reconstruction showed maxillary hyperplasia, malocclusion, and post-osteotomy changes in zygomatic and mandibular bones. We noted that her left infraorbital rim was higher than the right, which could contribute to the left eye strabismus. Magnetic resonance (MR) demonstrated infiltration of the surrounding soft tissues. A left maxillary sinus cyst was noted. Slight asymmetry was observed in her left brain and ventricle, resulting in midline displacement (Fig. 1B). The patient reported no history of neurological events. After admitting, the patient underwent debulking surgery, during which resected adipose tissue was collected for histological examination and whole exon sequencing (WES). Hematoxylin-eosin staining revealed a diffuse infiltration of the adipose tissue (Fig. 1C). Immunohistochemical analysis demonstrated predominantly adipocytes and endothelial cells exhibiting positive expression of IDH1 and PIK3CA (Fig. 1G). The initial WES analysis identified the IDH1: c.91A > G (p.Ile31Val) variant, with a variant allele fraction (VAF) of 45.45%. Given the possibility of a low abundance of the PIK3CA variant, we performed panel sequencing, which achieved an average sequencing depth of 10,000 × and >98% coverage (Fig. 1I). This analysis revealed that the patient carried both the heterozygous PIK3CA: c.1258T > C (p.C420R) variant and the IDH1: c.91A > G (p.I31V) variant, with VAF of 21.27% and 50.10%, respectively. Sanger sequencing was performed to validate WES result (Fig. 1J). We further collected blood samples for sequencing, but no variants were detected, suggesting that this was somatic variants.

**Figure 1:**
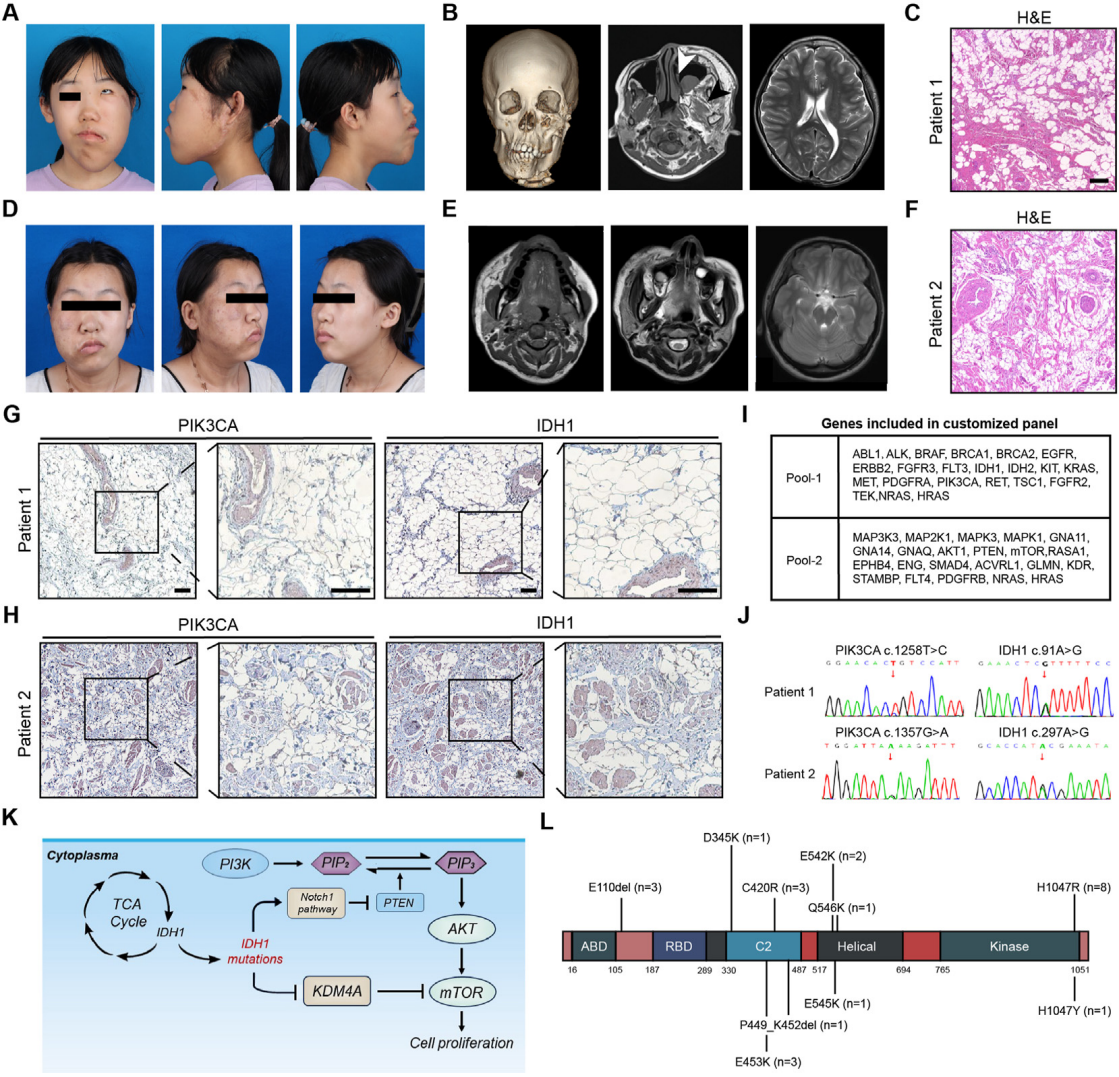
Patients' phenotypic features and potential molecular mechanism. **(A)**Appearance of patient No.1. The scar from the previous surgical intervention was prominently visible, and there were dispersed lesions resembling epidermal nevi (EN) located anterior to the scar. **(B)** CT skeletal reconstruction and MR images of patient No.1. Maxillary hyperplasia, malocclusion, and post-osteotomy changes in zygomatic and mandibular bones were observed. MR images showed the extensive adipose infiltrating of soft tissues, a maxillary sinus cyst, and hemimegalencephaly. **(C)** Hematoxylin and eosin staining on resected adipose tissue of patient No.1. Scale bar: 200 μm. **(D)** Appearance of patient No.2. Hyperpigmentation of the skin on the affected side of the face was noted. **(E)** MR image showed the enlargement and marginal infiltrating of masseter and parotid gland. The enlargement of parotid gland and cysts in the maxillary sinuses bilaterally was observed. MR images also revealed enlargement of right cerebellum. **(F)** Hematoxylin and eosin staining on biopsied sample of patient No.2. Scale bar: 200 μm. **(G)** Immunohistochemistry staining of PIK3CA and IDH1 on resected adipose tissue of patient No.1. Scale bar: 200 μm. **(H)** Immunohistochemistry staining of PIK3CA and IDH1 on biopsied sample of patient No.2. Scale bar: 200 μm. **(I)** Genes included in our customized panel. **(J)**Sanger sequencing validated the WES result. **(K)** Simplified schematic model for PI3K-AKT-mTOR pathway activation following IDH1 gain-of-function variants. **(L)** Overview of the distribution of PIK3CA mutant loci in 24 FIL patients without intracranial lesions.

Patient No.2, a 16-year-old female, was the first child of unrelated parents. Enlargement of the right face became evident gradually since birth. When she presented at our center, mild enlargement of the right cheek and right epidermal nevi (EN) were noted. The patient did not notice any differences in the facial skin on the right side until she was 13 years old, when a lesion resembling EN began to develop. The area of hyperpigmentation progressively expanded, accompanied by localized acne-like lesions. She had not received any medical interventions (Fig. 1D).

MR revealed enlargement of the masseter and parotid gland, with localized fatty infiltration at the margins. Bilateral cysts are observed in the maxillary sinuses. Additionally, we observed a larger cerebellum on the affected side, while there was no notable disparity in the cerebral hemispheres (Fig. 1E). The patient had no neurologic symptoms. Biopsy of the EN was collected for pathological staining and panel sequencing. Hematoxylin-eosin staining showed extensive infiltration of adipose tissue into the subcutaneous muscle layer (Fig. 1F), and Immunohistochemical analysis revealed high expression of IDH1 and PIK3CA in adipocytes and severed muscle fibers (Fig. 1H). The genetic analysis revealed the presence of a heterozygous PIK3CA: c.1357G > A (p.E453K) variant and a IDH1: c.297A > G (p. I99M) variant in EN, with VAF of 25.10% and 50.59%, respectively. No variants were detected in blood. The presence of the double variant was confirmed using Sanger sequencing (Fig. 1J). She is currently awaiting further surgery.

IDH1 is a crucial rate-limiting enzyme in the tricarboxylic acid cycle within the cytoplasm. It catalyzes the conversion of isocitrate into α-ketoglutarate (α-KG). However, IDH1 variants, such as the most common R132H variant, allow the reduction of α-KG to the (R)-2-hydroxyglutarate (2HG), which competitively inhibit the α-KG-dependent enzyme, further affecting epigenetic modification and cellular metabolism.^1^

Previous studies indicated that IDH1 variants may have a considerable impact on the regulation of the PI3K pathway. IDH1 variants have been shown to stimulate the Notch1 pathway and subsequently suppress Phosphatase and tensin homolog expression, thereby activating the PI3K pathway.^2^ Additionally, the 2-hydroxyglutarate metabolite produced by IDH1 variants can inhibit KDM4A and activate mTOR (Fig. 1K).^3^ However, most of the research was focused on the IDH1 R132H variant. Therefore, further basic experiments are needed to elucidate the relationship among the IDH1 variants identified in this study and the PI3K pathway.

IDH1 may play a role in the development of intracranial lesions as it is not only involved in driving the formation of gliomas, but also can impair differentiation by increasing apoptosis in various central nervous system lineages, leading to neuronal defects. Furthermore, IDH1 variants can interfere with cholesterol metabolism in astrocytes, and impact the brainstem and cerebellum, manifesting as gliomas in the posterior fossa.^4^ In fact, we have performed genetic testing on 24 FIL patients without concomitant intracranial lesions. PIK3CA variants were found in all the patients, including 8 distinct missense mutations and 2 frameshift mutations (Fig. 1L), but no IDH1 variants were identified. Limitations in brain tissue sampling impeded our exploration into whether facial and intracranial lesions share the same molecular variants. However, given that both the maxillofacial soft tissues and the brain originate from neural crest cells, it seems plausible to postulate that IDH1 variants produced within neural crest cells could exert influence on both the brain and facial soft tissues during early embryonic development.

It is essential to distinguish the second case from neurofibromatosis (NF). NF often presents with café-au-lait spots, cutaneous neurofibromas and Lisch nodules, and genetic testing often indicates NF1 mutation. Histologically, NF is mainly composed of nerve sheath cells, fibroblasts, muscle cells, and mucilage. Therefore, we have ruled out the diagnosis of NF for the second case. The differential diagnosis of FIL also includes other conditions. Hemangioma or vascular malformations can also cause facial swelling, which can be identified using MR. Other diseases with fatty infiltration, such as well-differentiated liposarcoma or lipoblastomatosis, can be excluded by pathological examination. Finally, conditions leading to contralateral hypoplasia, such as hemifacial microsomia or progressive hemifacial atrophy, should also be considered.

The advent of targeted therapies like alpelisib, a PI3K inhibitor, has marked a significant development in the management of overgrowth disorders. A FIL patient experienced a decrease in hyperplastic fat volume after treatment with alpelisib.^5^ However, the discovery of double variants poses an inherent challenge to these therapies, which are primarily designed to target a single molecular variant. Consequently, the discovery of this study underscores the pivotal role of multigene testing, which could assist in identifying all pertinent genetic alterations. This, in turn, could also facilitate the development of more accurate and efficacious therapeutic strategies.

In summary, this is the first time that both PIK3CA and IDH1 variants have been identified in patients with FIL. The findings are helpful to broaden our understanding of FIL, suggesting that we should pay attention to the pathogenic effect of additional variants.

## Ethics declaration

This study was approved by the Ethics Board of Shanghai Ninth Hospital, Shanghai Jiaotong University of Medicine (No. SH9H-2021-C46). Diagnostic genetic testing was performed after written informed consent received from the patients' parents, in accordance with local regulations. Written informed consent was obtained from both patients’ parents for the publication of clinical information and patient photographs.

## Conflict of interests

All authors declare that there are no competing interests.

## Funding

This work was supported by the Major and Key Cultivation Projects of Ninth People's Hospital affiliated to Shanghai Jiao Tong University School of Medicine (China) (No. JYZP005), the Treatment and Mechanism of PI3K/mTOR Dual-target Inhibitor (WX390) on PIK3CA-related Overgrowth Spectrum (PROS) (China) (No. JYWO22075) and 10.13039/501100012226Fundamental Research Funds for the Central Universities (China) (No. YG2023ZD13).
