# Supplementary material for: Concurrent PIK3CA and IDH1 variants in facial infiltrating lipomatosis with intracranial lesions
Source: Genes Dis. 2024 May 22;12(2):101324. doi: 10.1016/j.gendis.2024.101324 (PMC11614789; doi:10.1016/j.gendis.2024.101324)
Supplement: Multimedia component 1 [file mmc1.docx]

**Concurrent PIK3CA and IDH1 Variants in Facial Infiltrating Lipomatosis with Intracranial Lesions**

**Materials & Methods**

**Imaging modality**

MR examinations were performed using a MAGNETOM Verio with a magnetic field strength of 3.0 Tesla. The imaging protocol for maxillofacial MR included axial and coronal T1-weighted images (T1WI), axial and coronal T2-weighted images (T2WI), and axial and coronal T1-weighted images with fat suppression post-contrast. The cranial MR protocol included axial and sagittal T1WI, axial T2WI, and axial FLAIR sequences.

**Histopathological staining**

The samples were fixed in 10% (v/v) formalin, followed by dehydration in a series of gradient solutions. The fixed samples were then dehydrated in a gradient alcohol series. The tissue was washed in xylene and embedded in paraffin. The samples were sliced into sections approximately 5 μm thick and stained with haematoxylin and eosin.

**Immunohistochemical staining**

For immunohistochemistry, tissue sections were placed in a retrieval box filled with citrate buffer (pH 6.0) for antigen retrieval. The slides were washed in PBS on a decolorizing shaker. The sections were then incubated in a 3% hydrogen peroxide solution in the dark at room temperature for 25 minutes, followed by washing with PBS. Blocking was performed with 3% BSA for 30 minutes. The sections were then incubated with the anti-PIK3CA (Cell signaling technology, 4249T) or anti-IDH1 antibody (Proteintech, USA, 12332-1-AP) at 4°C overnight, followed by coincubation with the HRP-conjugated anti-rabbit IgG (Abcam, ab270144) for 1 h.

**DNA extraction and next-generation sequencing (NGS)**

We performed targeted sequencing using a high-depth NGS approach. The DNA was extracted from cells using the Qiagen DNA Extraction Kit (Qiagen, #13323). Genomic DNA fragments were spliced and modified for sequencing with the NEBNextillinautraII DNA Library Preparation Kit. After library establishment was completed, high-throughput sequencing was performed using the Illumina Nova Seq 6000 platform. The NGS panel had an average sequencing depth of 10,000X and 98% coverage. DNA sequences from the assay samples were compared to the reference sequence hg19 (GRCh37) and analyzed to determine the possible mutations.

**Sanger sequencing**

Conventional PCR was performed using a Veriti thermocycler (Applied Biosystems, Thermo Fisher Scientific MA). Amplified PCR products were analyzed by Tsingke (Shanghai, China) for Sanger sequencing.
